# Female Mice Are Not More Variable Than Males: Evidence From Continuous Glucose Monitoring in Normoglycaemic C57BL/6 Mice

**DOI:** 10.1111/dom.70639

**Published:** 2026-03-10

**Authors:** Matilda Kennard, Lydia F. Daniels Gatward, Manasi Nandi, Aileen J. F. King

**Affiliations:** ^1^ King's College London London UK

**Keywords:** animal pharmacology, continuous glucose monitoring (CGM), glycaemic control, mouse model

## 1 Introduction

Female mice are often excluded from preclinical studies across various scientific disciplines, including metabolic research, owing to the perception that the estrous cycle introduces variability in physiological readouts [1]. Murine estrous lasts 4–5 days and is characterised by fluctuations in oestrogen and progesterone concentrations; blood oestrogen levels are highest during the proestrus‐estrous (P‐E) stage, whilst levels of progesterone peak during the metestrus‐diestrous (M‐D) phase [2]. These hormones have been reported to influence blood glucose concentrations, and thus it is perhaps unsurprising that historically female mice have been excluded from preclinical metabolic research as researchers strive to minimise variability in experimental outputs which makes detecting treatment effects more challenging [1, 3]. However, there is a growing consensus that exclusion on the grounds of hormone fluctuations increasing variability may not be justified since studies have found no increased variability in numerous other physiological biomarkers in female rodents compared to males [4].

Glycaemic variability is a key indicator of overall glycaemic control. Unlike in humans, this is difficult to measure in mice as the standard method for measuring blood glucose relies on single time‐point glucometer measurements, which provide a snapshot of glycaemic control at a pre‐determined time. A recent advance in preclinical diabetes research has been the advent of continuous glucose monitoring (CGM) via radiotelemetry for rodent studies [5]. This allows for continuous measurement of blood glucose concentrations over several weeks in unrestrained mice. Here we use CGM to measure glycaemic variability in normoglycemic male and normoglycemic female mice at different estrous stages.

## 2 Methods

### 2.1 Animals

Female (*n* = 7) and male (*n* = 11) 10–16‐week‐old C57BL/6J mice were implanted with HD‐XG telemetry devices in the aortic arch under general anaesthetic (Data Sciences International, USA) (Figure S1) [5]. Following surgery recovery (1‐week), blood glucose concentrations were recorded every 1‐s and averaged every 10‐s for up to 8‐weeks. 2–25 days per mouse (mean of 14 days) over this time span were considered ‘undisturbed days’ when no other experimentation occurred and this data was used to investigate glycaemic variability. Data from these days were separated into light/day (7 am–7 pm) and dark/night (7 pm–7 am) periods due to known differences in food intake, activity and blood glucose concentrations linked to circadian rhythms [6]. Mice implanted with telemetry probes were housed with a non‐surgical ‘buddy’. All animals were housed in a controlled environment with *ad libitum* access to food and water.

### 2.2 Measures of Glycaemic Variability

Glycaemic variability was quantified using several methods previously defined [7, 8]. Within‐day glycaemic variability was calculated by various measures. Mean absolute deviation (MAD), which calculates the average deviation of each datapoint from the mean for each 12 h cycle, was used to determine second‐to‐second variation in glucose concentrations. Continuous overall net glycaemic action (CONGA‐2), which calculates the difference between blood glucose values a set time apart (e.g., CONGA‐2 = every 2 h) and the standard deviation of these differences, was used to determine hour‐to‐hour glycaemic variation. Mean amplitude of glycaemic excursions (MAGE), which calculates the average size of deviations, which are at least 1 standard deviation from the mean, was used to determine larger fluctuations in glucose concentrations such as those associated with food intake. Between‐day variability was determined using mean of daily differences (MODD) which determines differences in glucose values at the same timepoint on different days. Between‐animal variability was determined using coefficient of variation (CV) which calculates how far each mouse's glucose value deviated from the cohort mean on each day.

### 2.3 Estrous Staging

Vaginal smears were stained with methylene blue and estrous stage was determined based on proportions of nucleated epithelium, cornified epithelium and leukocytes, and were assigned ‘proestrus‐estrous’ (P‐E) or ‘metestrus‐diestrous’ (M‐D). Before each day where blood glucose concentrations were recorded in undisturbed mice, smears were taken for the preceding 3‐days to determine estrous stage on the recorded day.

### 2.4 Statistical Analysis

Unpaired *t*‐tests were used to compare two groups. ANOVA with Holm Sidak post hoc test was used to compare multiple groups. Statistical analysis was conducted in SigmaPlot 14.0 and graphs were plotted in GraphPad prism.

### 2.5 Ethics Statement

All in vivo procedures were approved by our institution's ethics committee and performed under licence in accordance with the U.K. Home Office Animals (Scientific Procedures) Act 1986 with 2012 amendments.

## 3 Results

**FIGURE 1:**
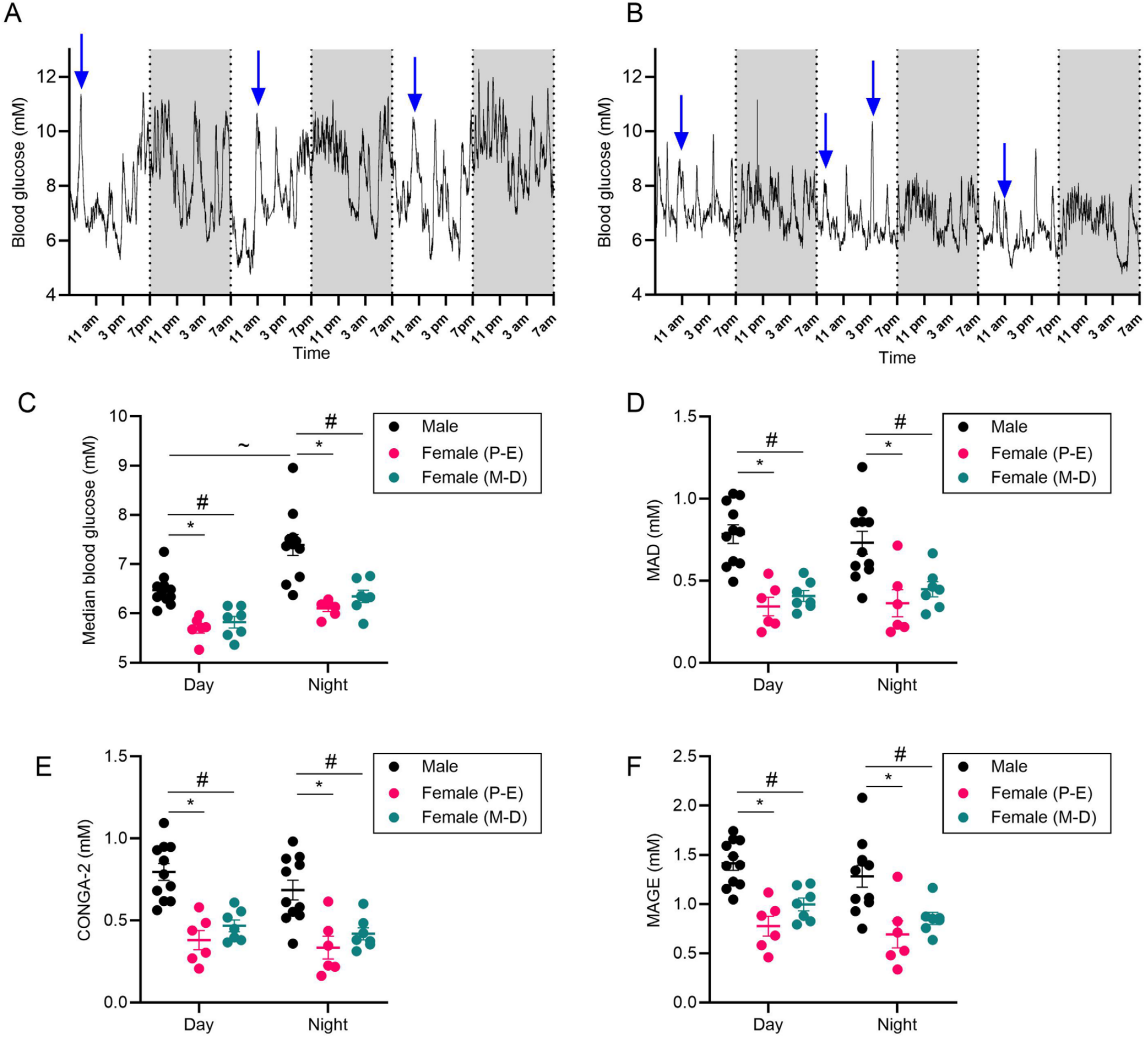
Diurnal rhythms and sex differences in blood glucose concentrations and glycaemic variability in male (average of *n* = 11) and female (*n* = 6–7) normoglycaemic mice. (A, B) 10 s average blood glucose concentrations over 72 h in a representative (A) male and (B) female mouse. Blue arrows represent times of animal unit staff entering the room for welfare checks. (C) Overall median blood glucose concentrations, (D) mean absolute deviation (MAD), (E) continuous overall net glycaemic action 2 (CONGA‐2) and (F) mean amplitude of glycaemic excursions (MAGE) for the entire experimentation in males, females in proestrus‐estrous (P‐E) and females in metestrus‐diestrous (M‐D) during the day (07:00–18:59) and night (19:00–06:59).* and # represent a significant difference compared to females in P‐E and females in M‐D, respectively. ~ represents a significant difference compared to the daytime. *p* < 0.05, two‐way ANOVA with Holm‐Sidak post hoc test, *n* = 6–11 animals. In scatter plots, data represents the mean ± SEM of individual mice averages across 2–25 days.

Overall, female mice had lower blood glucose concentrations compared to males irrespective of estrous stage and both during the day (males: 6.5 ± 0.1 mM; females in P‐E: 5.7 ± 0.1 mM; females in M‐D: 5.8 ± 0.1 mM; *p* < 0.05) and at night (males: 7.4 ± 0.2 mM; females in P‐E: 6.1 ± 0.1 mM; females in M‐D: 6.3 ± 0.1 mM; *p* < 0.05) (Figure 1C). Interestingly, whilst male blood glucose concentrations were significantly higher during the night vs. day, female day vs. night blood glucose concentrations were comparable.

Blood glucose variability across the day and night (within‐day) was measured across the whole experiment using MAD, CONGA‐2 and MAGE. All three measures of variability suggest that female mice exhibit reduced variability in blood glucose concentration compared to males both during the night and during the day, with variability typically 2‐fold lower irrespective of estrous stage in the female mice (Figure 1D–F). Although female mice in the metestrus‐diestrous stage showed a trend for increased glycaemic variability compared to proestrus‐estrous, this was not significant for any measures of variability.

**FIGURE 2:**
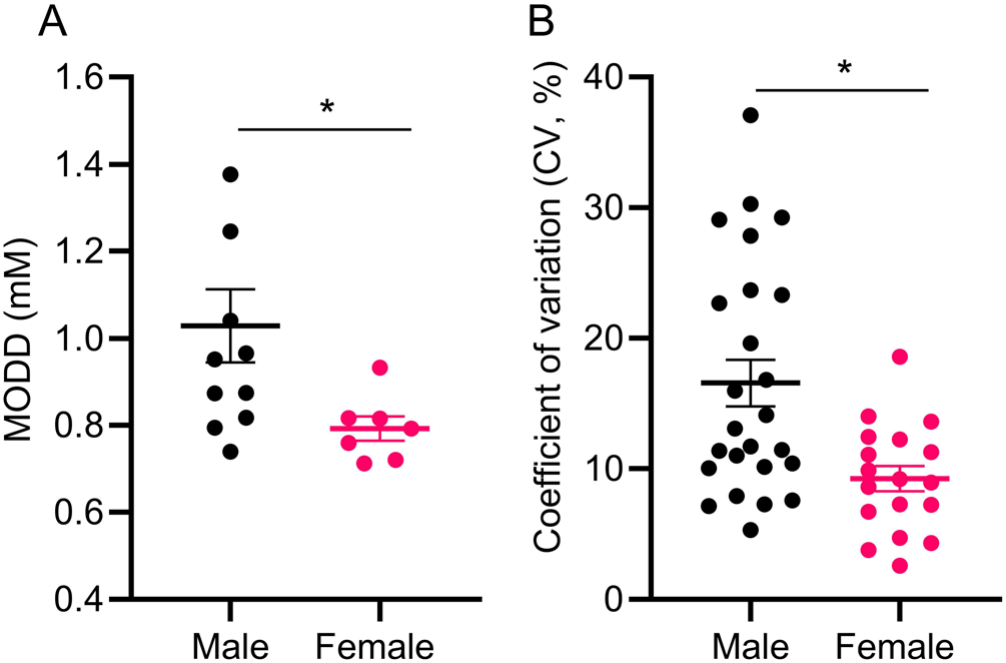
(A) Between‐day variability, as measured by mean of daily differences (MODD), in male and female normoglycaemic mice. Data represents mean ± SEM of individual mouse averages across 2–25 days (*n* = 7–11 mice). (B) Between‐animal variability, as measured by coefficient of variation (CV) for all mice vs. the cohort average, in males and females. Data represents mean ± SEM for individual days in all mice (*n* = 18–25 days). *p < 0.05, unpaired *t*‐test.

In line with these findings, day‐to‐day (between‐day) variability calculated using MODD was higher in male mice despite female mice cycling through the four estrous stages across days (male: 1.03 ± 0.08 vs. female: 0.79 ± 0.03 mM; *p* < 0.05) (Figure 2A). Furthermore, between‐animal variability, calculated using coefficient of variation (CV), was also higher in male mice (Figure 2B, male: 15.1% ± 1.06% vs. female: 9.5% ± 0.99%; *p* < 0.01).

## 4 Discussion

CGM in unrestrained mice has allowed us to capture and quantify variability in glycaemic control. This is impossible to achieve with standard methods of measuring glucose concentration using handheld glucometers. CGM has highlighted that female mice do not exhibit higher blood glucose variability compared to male mice. Although female mice in the metestrus‐diestrous phase (when oestrogen levels are low and progesterone levels are high) tended to have slightly higher median blood glucose concentrations and within‐day glycaemic variability, these differences were not significant and did not supersede the higher variability detected in male mice. In essence, the field has often excluded female mice on the basis that the estrous cycle introduces excessive variability, while overlooking the substantial variability already present in male mice. Other studies, including those from our group, have highlighted the importance of using both male and female mice in metabolic research but we, for the first time, provide empirical evidence that challenges the concept that the estrous cycle adds an unacceptable level of variation [5, 9].

This study was performed in normoglycaemic mice, and while future work should extend these assessments to diabetic models, our findings provide a clear indication that estrous‐related variability is not a major driver of glycaemic variation under baseline conditions. Diabetes is defined by an inability to tightly regulate blood glucose concentrations and therefore diabetic models naturally display more extreme glucose fluctuations. However, this greater variability is generally not regarded as a limitation when using male mice, which are often selected precisely because they develop more severe metabolic phenotypes. This inconsistency in when variability is considered acceptable reinforces that estrous‐related fluctuations are not a scientifically sound reason to exclude females from preclinical metabolic research, irrespective of the model. There is a persistent misconception among researchers that including female mice will double the number of animals required. However, there is evidence that by analysing data using appropriate factorial designs, incorporating both sexes does not require doubling sample size [10].

Sex differences in phenotype severity have also contributed to the historical exclusion of female mice from preclinical metabolic research [11]. This can be overcome by protocol adjustment or by using female mice to justifiably also model a milder phenotype [12].

In conclusion, female mice do not show greater glycaemic variability than males, indicating that estrous cycling is not a legitimate reason for their exclusion from metabolic studies.

## Author Contributions

M.K., M.N. and A.J.F.K. contributed to the conception, design, acquisition and interpretation of data. M.K. and L.F.D.G. drafted the article. All authors contributed to the interpretation of the results and gave final approval of the report to be published.

## Funding

This project was funded by the British Pharmacology Society.

## Conflicts of Interest

The authors declare no conflicts of interest.

## Ethics Statement

The authors have nothing to report.

## Supporting information


**Appendix S1:** dom‐26‐0400‐reslet‐File005.docx.
**Figure S1:** Schematic outlining experimental design including experimental outcomes.
**Figure S2:** Body weight monitoring of male (A) and female (B) mice implanted with telemetry probes and non‐implanted cage mate ‘buddies’. * = significant difference between telemetry and buddy mice, # = significant difference compared to day 0. *p* < 0.05, two‐way repeated measure ANOVA with Bonferroni post hoc test, *n* = 7–11 mice. Data are mean ± SEM.

## Data Availability

Data is available upon request from A. J. F. King: aileen.king@kcl.ac.uk.
