# Supplementary material for: Female Mice Are Not More Variable Than Males: Evidence From Continuous Glucose Monitoring in Normoglycaemic C57BL/6 Mice
Source: Diabetes Obes Metab. 2026 Mar 10;28(6):5343–6. doi: 10.1111/dom.70639 (PMC13146135; doi:10.1111/dom.70639)
Supplement: Supplementary file 1 — Appendix S1: dom‐26‐0400‐reslet‐File005.docx. Figure S1: Schematic outlining experimental design including experimental outcomes. Figure S2: Body weight monitoring of male (A) and female (B) mice implanted with telemetry probes and non‐implanted cage mate ‘buddies’. * = significant difference between telemetry and buddy mice, # = significant difference compared to day 0. p < 0.05, two‐way repeated measure ANOVA with Bonferroni post hoc test, n = 7–11 mice. Data are mean ± SEM. [file DOM-28-5343-s001.docx]

Methods:

**Experimental procedures: Implantation of telemetry probes**

Telemetry probes (HD-XG glucose implant, Data Sciences International) were implanted into the aortic arch with the transmitter portion placed subcutaneously. Briefly carprofen was administered intraperitoneally (4mg/kg) prior to surgery. Isoflurane was then used to anesthetise the animal before the neck region was shaved and cleaned with surgical scrub and ethanol, and eye lubrication was applied to the eyes. Lack of pedal reflexes and breathing was used to confirm appropriate anaesthetic depth. With the mouse in the supine position and head facing towards the surgeon, a midline incision (1-2cm) was made to the neck, and the salivary glands were separated gently by blunt dissection. The left carotid artery was located, gently separated from the vagus nerve and tied off at the most cranial end using non-absorbable suture. A loose suture was also used to occlude blood flow through the vessel at the end proximal to the heart. The occluded vessel was then punctured using a 26G needle and needle holders were used to introduce the glucose sensing portion of the device into the vessel, this was then progressed until the tip of the sensor resided in the aortic arch (facilitated by marker bands on the sensor itself). Suture was then tied to secure the sensor in place and a subcutaneous pocket down the right flank of the animal (made via the initial incision) was made to accommodate the transmitter portion of the device. Suture was used to close the initial incision and Marcain (2mg/kg) was provided subcutaneously proximal to the incision for pain relief.

The animal was recovered in a warming cabinet and closely observed. Once the animal recovered from anaesthetic and was seen to be performing expected activities (eating, grooming, reaching up) the mouse was moved to its home cage and allowed to recover for 7 days prior to experimental manipulations. 24-hours post-surgery 4mg/kg carprofen was administered intraperitoneally. Mice were monitored daily to ensure appropriate recovery in this period. Surgery was performed in the morning to allow time for appropriate surgical recovery. Anaesthetic and analgesic regime was chosen after consultation with a veterinary surgeon.

**Experimental animals:**

The wildtype C57BL6/J mice used in this study were sourced from Charles River, they had a median age of 11 weeks at time of telemetry implantation and an age range of 10-16 weeks. Data for this study was recorded on days where mice were ‘undisturbed’ which equated to 2-25 days per animal (mean number of days per animal was 14-days) over up to an 8-week period depending on probe lifespan. On the remaining days procedures including glucose tolerance testing and tail prick for handheld glucometer measurements of blood glucose concentration were taken for calibration purposes. Whole cage changes, fasting (up to 6h) and handling were also carried out on the remaining ‘disturbed’ days. Non transplanted cage buddy mice were sex and aged-matched.

**Housing and husbandry:**

Mice were housed in a conventional unit in open-top cages. They had *ad libitum* access to chow (standard rodent diet 20, Picolab UK) and water. For 3-days following surgical recovery, mice were fed a mash made from standard chow. Bedding material consisted of paper and woodchip and enrichment included cardboard tunnels. Temperature in the animal facility was maintained in a narrow range (20-24 °C) and humidity was 45-65%. Mice were on a 7am-7pm light/dark cycle. Welfare checks were carried out at least once a day.

**Sample size:**

The study was designed to characterise sex differences in blood glucose variability. At the time of study design, reliable sex-specific variance estimates for telemetry-derived measures of glycaemic variability were not available to support formal power calculations. Initial sample sizes were therefore determined based on feasibility and prior laboratory experience with male mice in diabetes and telemetry models, resulting in a modestly larger male cohort. Females were included specifically to enable assessment of sex as a biological variable.

**Allocating animals/samples to experimental groups**

Females were separated into groups depending on estrous stage ascertained through vaginal swabs. The order in which surgery was carried out was randomised but only females over 23g and males over 26g were selected for telemetry probe intervention since Data Science International report better surgery success in mice of higher body weights.

**Experimental outcomes:**

Experimental outcomes were median blood glucose concentrations and glycaemic variability (MAD, CONGA-2, MAGE, MODD, CV).


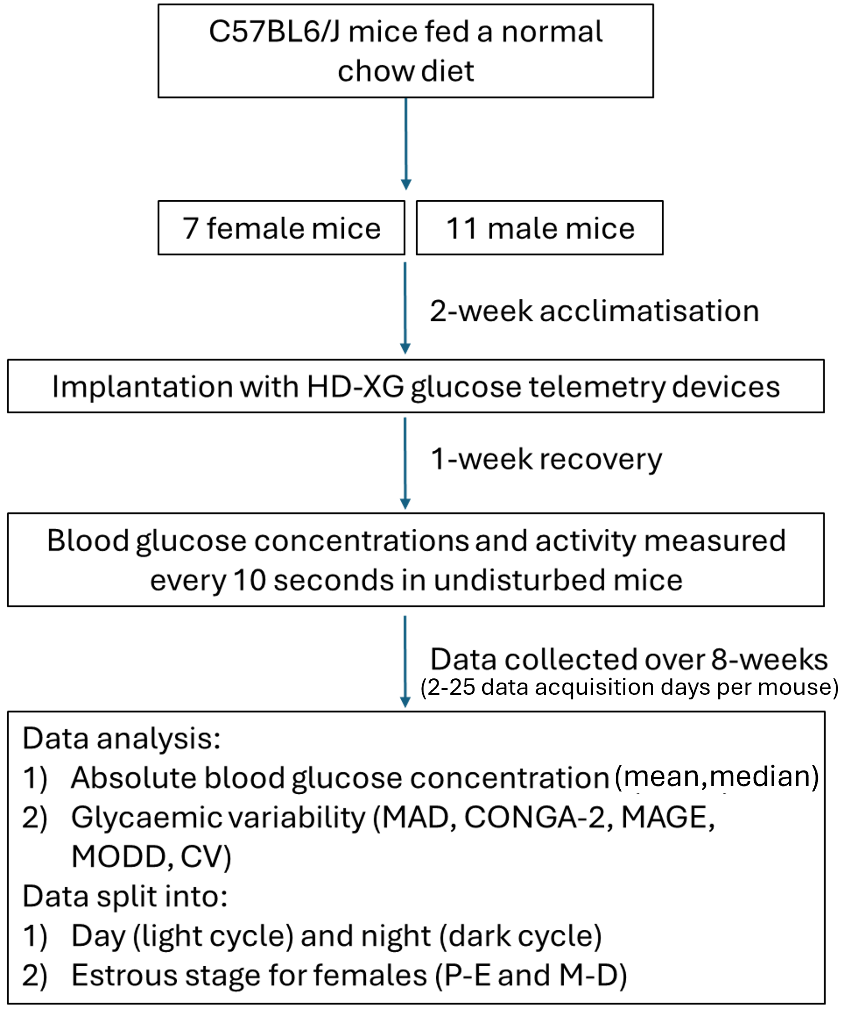


***Figure 1:*** *Schematic outlining experimental design including experimental outcomes.*

Results:

**Baseline data:**

The maximum weight loss in a single mouse caused by surgical implantation of the telemetry probe was 3.6g for males and 2.7g for females. In both male and female mice body weight was no different from pre-surgery (0 days) by 7-days post-surgery (figure 2).


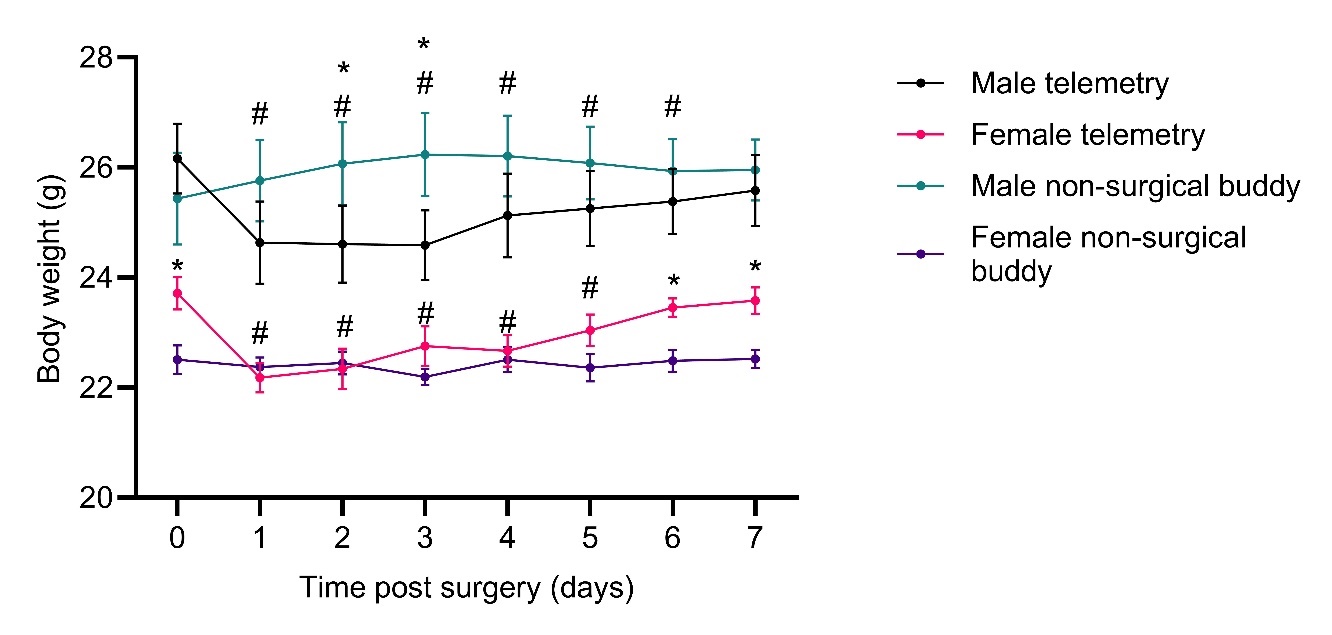


***Figure 2:*** *Body weight monitoring of male (A) and female (B) mice implanted with telemetry probes and non-implanted cage mate ‘buddies’. * = significant difference between telemetry and buddy mice, #= significant difference compared to day 0. P<0.05, two-way repeated measure ANOVA with Bonferroni post-hoc test, n=7-11 mice. Data are mean±SEM.*

**Adverse events:**

One male and one female were withdrawn within one week of surgery due to post-operative welfare concerns (lethargy, reduced mobility, piloerection), resulting in final group sizes of 11 males and 7 females. Attrition was balanced across sexes and unrelated to experimental outcomes. Since this adverse event impacted a small proportion of the animals, no modifications were made to reduce adverse events in this case. 1 female was also withdrawn mid-way through the study due to welfare concerns and thus only 2 undisturbed days were recorded for this mouse.
